# Weight change and the risk of incident atrial fibrillation: a systematic review and meta-analysis

**DOI:** 10.1136/heartjnl-2019-314931

**Published:** 2019-06-22

**Authors:** Nicholas R Jones, Kathryn S Taylor, Clare, J Taylor, Paul Aveyard

**Affiliations:** Nuffield Department of Primary Care Health Sciences, University of Oxford, Oxford, UK

**Keywords:** Atrial Fibrillation, Systemic Review, Meta-analysis, Obesity, Global Disease Patterns

## Abstract

**Background:**

The prevalence of obesity is increasing globally and this could partly explain the worldwide increase in the prevalence of atrial fibrillation (AF), as both overweight and obesity are established risk factors. However, the relationship between weight change and risk of incident AF, independent of starting weight, remains uncertain.

**Methods:**

MEDLINE, Embase, Pubmed, Web of Science, Cochrane Central Register of Controlled Trials, Database of Abstracts of Reviews of Effects, Trials Register—clinicaltrials.gov, CINAHL and the WHO ICTRP were searched from inception to July 2018.

We included randomised controlled trials and cohort studies across all healthcare settings but excluded studies of bariatric surgery. A random effects model was used to calculate pooled hazard ratios. The primary outcome was the risk of incident AF in relation to weight change.

**Results:**

Ten studies, including 108 996 people, met our inclusion criteria. For a 5% gain in weight, the incidence of AF increased by 13% (HR 1.13, 95% CI 1.04 to 1.23, I^2^=70%, n>20 411 in five studies; study size was unknown for one study). A 5% loss in body weight was not associated with a significant change in the incidence of AF (HR 1.04, 95% CI 0.94 to 1.16, I^2^=73%, n=40 704 in five studies).

**Conclusions:**

Weight gain may increase the risk of AF, but there was no clear evidence that non-surgical weight loss altered AF incidence. Strategies to prevent weight gain in the population may reduce the global burden of AF. Given the lack of studies and methodological limitations, further research is needed.

## Background

Atrial fibrillation (AF) is the most common sustained cardiac arrhythmia, affecting 33.5 million people worldwide.^1^ It is associated with a fivefold increased risk of embolic stroke, 50% increased risk of death in the 3 years following diagnosis and reduced quality of life.^2–4^ AF is also costly, accounting for 2% of healthcare expenditure in high-income countries.^5^

The prevalence of AF is rising and anticipated to double in Europe before 2060 due to an ageing population and increased prevalence of established risk factors including obesity, hypertension, diabetes and obstructive sleep apnoea.^6^ Three systematic reviews have demonstrated that obesity is associated with an increased risk of incident AF.^7–9^ The pathological mechanisms linking excess weight and AF are not fully understood but may be mediated by the metabolic syndrome and direct damage to the heart from excess adipose tissue. Overweight leads to an increase in epicardial adipose tissue, atrial enlargement and diastolic dysfunction, which can lead to atrial electrical remodelling and an increased risk of AF.^10^

In people diagnosed with AF, increasing body mass index (BMI) and pericardial fat volumes are associated with a higher symptom burden.^11 12^ Weight reduction and effective risk factor management in overweight individuals can reduce the frequency and duration of AF episodes and increase arrhythmia-free survival postablation.^13^ However, whether weight change alters the risk of developing AF is uncertain. The aim of this study was to assess the association between weight change and AF incidence and, if possible, determine whether the association was modified by baseline weight status.

## Methods

The review was conducted following the Preferred Reporting Items for Systematic Reviews and Meta-analyses (PRISMA) guidelines (eAppendix 1). Our methods were prespecified in our study protocol, which is included in the online supplementary file (eMethods 1).

10.1136/heartjnl-2019-314931.supp1 Supplementary file 1

10.1136/heartjnl-2019-314931.supp8 Supplementary file 8

### Search strategy

We searched nine electronic databases from inception to July 2018 (eMethods 2). One author (NJ) screened all the abstracts and titles, and two authors screened the full texts (NJ, PA and KT (in pairs)). We manually checked the references of the full-text papers to ensure literature saturation.

10.1136/heartjnl-2019-314931.supp2 Supplementary file 2

### Eligibility criteria

We included randomised controlled trials (RCTs) and cohort studies of weight change (measured by kg, BMI or percentage weight change) in adults who reported the incidence of paroxysmal, persistent or permanent AF as an outcome. There was no restriction by language or setting.

We excluded case–control studies, case reports and case series, as they could not assess the incidence of AF, and conference abstracts as they provided insufficient information to appraise the study. We excluded studies of bariatric surgery, as the rapid, large volume of weight loss along with nutritional and physiological differences induced by the surgery may not reflect the association between weight change and AF in the general population.

For each study, two authors (NJ and KT) independently extracted data, contacting authors as necessary.

### Assessment of risk of bias and quality of the evidence

Two authors (NJ and KT) independently assessed the risk of bias using the Newcastle-Ottawa Scale (NOS) for non-randomised studies.^14^ Two authors (NJ and PA) independently assessed the risk of bias and strength of evidence across the studies, using the Grading of Recommendations Assessment, Development and Evaluation (GRADE) framework headings.^15^ A full GRADE assessment was precluded because not all studies reported the absolute number of people with incident AF in each weight change category. For each assessment, disagreements were discussed and resolved.

### Data analysis and synthesis

We log-transformed the reported HRs across the weight change categories and calculated study-specific slopes (linear trends) using the method of Greenland and Longnecker and the Stata glst command of Orsini *et al*.^16 17^ Where possible, we undertook random effects meta-analysis based on the DerSimonian and Laird method to compute the summary HRs with 95% CIs of incident AF.^18^ We assessed statistical heterogeneity using the I-squared statistic and calculated approximate 95% prediction intervals to estimate the likely effect in an individual setting using the methods of Higgins *et al*.^19 20^ Where there were insufficient data to pool, we report results descriptively.

We could not carry out our prespecified subgroup analyses (by intentionality of weight loss, initial weight, comorbidity and gender) and instead, we subgrouped by population type and duration of follow-up in a post hoc analysis. We assessed whether the results were sensitive to the choice of model, quality of the studies, whether data were estimated from the presented results and the heterogeneity of the patient populations. To do so, with separate analyses, we carried out fixed-effects analysis and excluded individual studies, studies with poor quality data and those for which we estimated data. There were insufficient data to carry out meta-regression or assess publication bias (exploratory contour plots are included in the online supplementary file (eMethods 3). All analyses were carried out using Stata V.14.2.

10.1136/heartjnl-2019-314931.supp3 Supplementary file 3 method3

## Results

### Study characteristics

**Figure 1:**
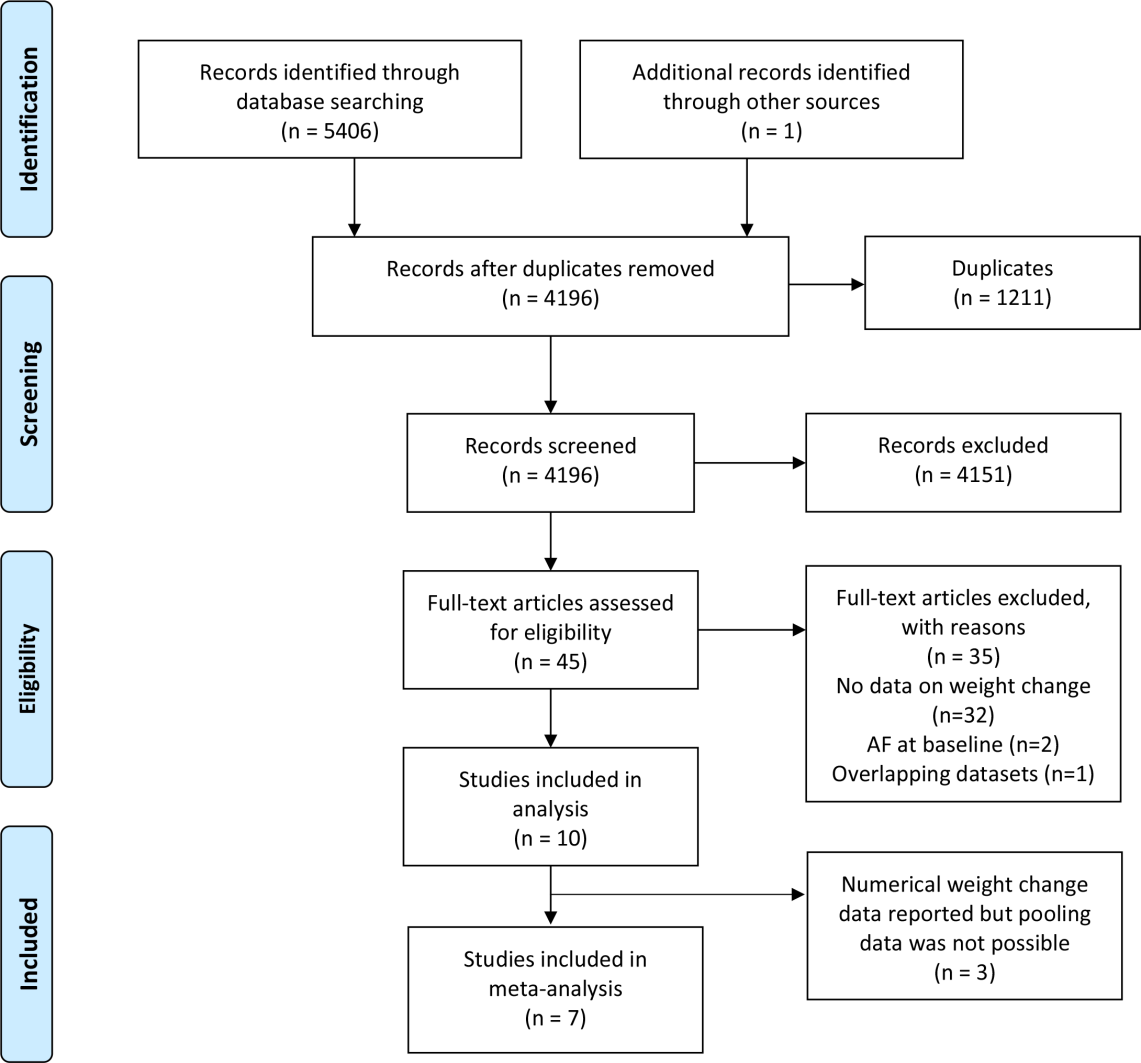
Preferred Reporting Items for Systematic Reviews and Meta-analyses flow diagram of study selection. AF, atrial fibrillation.

From 5407 records, we screened 45 full texts, of which 10 met the eligibility criteria (figure 1). Nine were cohort studies (table 1). In the single RCT, Alonso *et al* randomised participants with type 2 diabetes (T2DM) to an intensive weight loss intervention or a diabetes support and education programme.^21^ We included the combined data for these two groups, reported in a multivariate analysis adjusted for a range of important covariates including age, blood pressure and intervention arm (eTable 1).

10.1136/heartjnl-2019-314931.supp4 Supplementary file 4 table1

**Table 1:** Characteristics of participants in the included studies

First author (year)	Population	Total population (analysis population)	Age mean (SD)	Female (%)	Study population baseline weight status			
					Mean BMI or weight (weight ranges)	Healthy BMI (%)	Overweight (%)	Obese (%)
Rosengren *et al* 29	Men born in Goteborg, Sweden, between 1915 and 1925 participating in the Primary Prevention Study.	6903 (6412)	51.5 (2.3)	0%	Midlife BMI 25.6	46	46	8
Tedrow *et al* 24	Female health professionals with no prior CVD who had participated in the Women’s Health Study (USA), a RCT of aspirin versus vitamin E.	34 309	54.6 (7.0)	100%	Mean weight 70.2 kg	51	31	18
Grundvold *et al* 23	Prospective CVD survey of healthy male employees from five government institutions in Oslo, Norway, aged 40–59 years.	2014 (1997)	50.0 (5.5)	0%	Mean BMI 25	60	36	4
Huxley *et al* 30	Participants of the Atherosclerosis Risk in Communities Study, from four study sites across the USA.	14 219 (10 239)	54.2 (5.7)	55%	Mean BMI 27.8	33	40	27
Alonso *et al* 21	The Look AHEAD trial (USA) randomised people aged 45–76 with T2DM to an intensive lifestyle intervention versus diabetes support and education.	5067 (4906)*	59.0 (7.0)	60%	Mean BMI 36.0	0	100	
Grundvold *et al* 22	People with newly diagnosed T2DM in Sweden.	7169	59.5 (10.3)	47%	Mean BMI 31.1	10	36	54
Johnson *et al* 25	Middle-aged men in Swedish health screening programme.	5633	47.0 (3.0)	0%	Mean weight 77.1 kg	57	43	
Berkovitch *et al* 28	Healthy people undergoing periodic private health screening in Israel.	18 290	49.0 (11)	27%	Mean BMI 26.1	42	44	14
Diouf *et al* 27	Respondents of the AusDiab general population survey of adults ≥25 years in Australia.	8273 (5389 who had follow-up ECG)	56.6 (11.7)	52%	Not stated	35†	42†	23†
Ball *et al* 26	Population longitudinal cohort study involving cardiovascular disease screening via health survey and periodic study visits—The Tromsø Study, Norway	14 652 (attended third and fourth survey and provided follow-up BMI data)	34.1 (17.6)	51%	Mean BMI 24.2	Not stated Not stated		

Data is given for the total population unless otherwise stated.

*Estimated.

†Based on 8214 of the 8273.

‡Additional 1% underweight at baseline. Figures are based on total fourth study baseline data.

AF, atrial fibrillation; BMI, body mass index; CVD, cardiovascular disease; RCT, randomised controlled trial; T2DM, type 2 diabetes mellitus.

In general, participants were middle-aged people attending routine health checks, but in two studies all participants had T2DM.^21 22^ AF incidence rates were higher in studies of people with diabetes or cardiovascular risk factors compared with population surveys of healthy people (table 1). In four studies, most participants had a healthy weight at baseline,^23–26^ whereas in six most were overweight or obese. Only the RCT of the weight loss programme assessed whether participants intended to lose weight.^21^ Follow-up varied between 4.6 and 35 years. Six studies reported the incidence of AF during follow-up after the period of measured weight change, whereas four reported the association between weight change and AF incidence across the same period (table 2). Nine studies used linked electronic health records to capture incident cases of AF (table 1). In four studies, this was triangulated with ECGs at study follow-up visits. One study relied on a single follow-up visit ECG to capture incident cases.^27^ In one study, AF was detected by 7 day Holter monitoring.^28^

**Table 2:** Follow-up methods, including weight assessments and AF case definition

First author (year)	Follow-up (years)	When weight change was assessed	How weight change was assessed	AF case definition	Relative timing of weight and AF assessment	AF incidence per 1000 person years
Rosengren *et al* 29	Maximum 34.3	Single midlife evaluation (at 12 years after screening appointment) where weight was measured and participants reported their recalled weight at age 20 years.	Weight change between recalled weight at age 20 and midlife evaluation (approximately 30-year interval).	Hospital discharge code for AF	Weight change calculated at single midlife evaluation visit (1970–1973). AF incidence recorded over subsequent study follow-up to 31 December 2004. Median time between midlife evaluation and discharge for AF was 25 years.	7.5
Tedrow *et al* 24	Mean 12.9	Self-reported weight on the 24, 36, 60, 72and 108 month questionnaires during the randomised trial, at the beginning of the observational phase and yearly thereafter. Self-reported height used to calculate BMI.	BMI change over the first 60 months of the study (5-year interval).	Participant self-reporting, triangulated against electronic health record	Weight change in first 60 months of study and AF incidence after that time period through to the end of the study.	1.9
Grundvold *et al* 23	Maximum 35	Measured weight at baseline visit and recalled weight at age 25 years recorded at that time. Baseline visits 1972 to 1975.	Weight change between recalled weight and baseline (approximately 25 year interval).	ECG at study visit and hospital discharge codes	Weight change calculated at baseline visit based on change from recalled weight aged 25 years. AF incidence over subsequent follow-up, up to 31 December 2007.	4.5
Huxley *et al* 30	Maximum 11	Weight change between baseline and visit four (9-year interval)	Weight was measured at baseline and at 3, 6 and 9 years later.	ECG at study visit and codes from hospital discharge and death certificates	Weight change and AF incident cases across same study follow-up period.	6.9
Alonso *et al* 21	Mean 9	Weight was measured at baseline and annually thereafter. Measurements done in duplicate using a digital scale and stadiometer.	Weight change was between baseline and first annual visit (1-year interval)	ECG at biannual follow-up visit and hospital discharge codes	Weight change in first year of study with AF incidence calculated across follow-up, including the first year.	6.4
Grundvold *et al* 22	Mean 4.6	Weight was measured at baseline and at a second visit within 18 months.	Weight change was between the two visits (average intervals were 421, 396 and 402 days for the weight gain, stable weight and weight loss categories, ie, around 13–14 months)	Codes from national patient registries and primary care electronic database	Follow-up for AF started after the second visit and continued until new AF diagnosis, death or 31 December 2009.	9.8*
Johnson *et al* 25	Mean 22.3 (from rescreening) 28.3 (from baseline)	Weight was measured at two screening examinations, separated by an average of 6 years (first 1974–1984, second 1981–1985).	Average annual weight change was calculated.	Hospital inpatient and outpatient codes	Weight change between first and second screening examinations, AF incidence between second screening examination and study endpoint, 31 December 2010.	8.5*
Berkovitch *et al* 28	Mean 6.4	Weight was measured at annual visits.	The weight change between visits was integrated into the Cox model as a time-dependent variable.	ECG at study visit, Holter monitor or primary care electronic records	Weight change and AF incidence both reported across same study follow-up period.	2.5*
Diouf *et al* 27	Mean 5	Weight was measured at baseline (in 1999/2000) and a second visit (in 2004/2005).	Weight change between the two visits (approximately 5-year interval)	Single study follow-up ECG	Weight change and AF incidence both reported across same study follow-up period.	2.0
Ball *et al* 26	Mean 15.7	BMI was measured at each study survey. Participants visited a study centre and were weighed in light clothing with no shoes for consistency.	Change in BMI between the third (1986–1987) and fourth (1994–1995) survey	Hospital inpatient and outpatient records, linked with National Causes of Death Registry	Weight change between third and fourth survey used to calculate BMI change. Incidence of AF calculated over subsequent follow-up after fourth survey to 31 December 2013.	3.6

AF, atrial fibrillation; BMI, body mass index.

### Weight gain and risk of incident AF

#### Percentage weight gain

**Figure 2:**
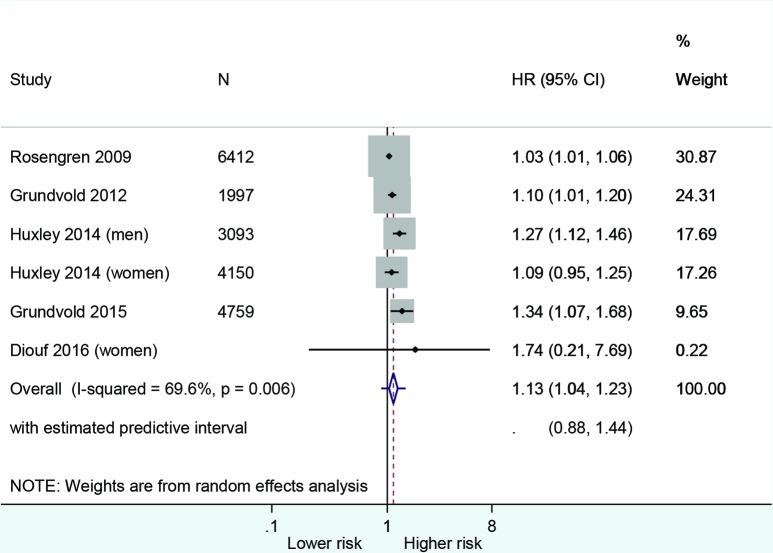
Risk of 5% weight gain on incidence of atrial fibrillation.

Pooled analysis from six populations (five studies) showed that for a 5% gain in weight, the incidence of AF increased by 13% (HR 1.13, 95% CI 1.04 to 1.23, with 95% prediction interval 0.88 to 1.44) (figure 2), with moderately high statistical heterogeneity. Excluding the study by Rosengren *et al* reduced the I^2^ statistic from 70% to 33%.^29^ Most participants were overweight or obese at the outset in five of the six populations.

Sensitivity analysis generally produced consistent results (eTable 2). Outcome data were converted to percentage weight gain for two studies included in the pooled analysis.^22 23^ Excluding these studies did not change the point estimate for incident AF but the result was no longer statistically significant (eTable 2). In both studies of general populations and in the single study of a population with T2DM,^22^ a 5% gain in weight was associated with a significant risk of AF, and the same result applied for both studies with follow-up less than and greater than 10 years (eTable 2).

10.1136/heartjnl-2019-314931.supp5 Supplementary file 5 table 2

#### Other measures of weight gain

Several studies reported weight gain in continuous or categorical terms by kg or BMI gained. Where possible, these results were converted and pooled.^22 23^ We were unable to pool data from three studies.^24–26^ Johnson *et al* reported the association between an annual increase in weight measured by kg in the first 6 years of follow-up and incidence of AF in the subsequent years of follow-up (mean 22.3 years).^25^ In this study the incidence of AF increased by 14% (HR 1.14, 95% CI 1.05 to 1.24) for a 1 kg gain per annum. Ball *et al* reported the association between weight gain and risk of AF by change in BMI category.^26^ A 4 kg/m^2^ increase in BMI was associated with a 35% increased risk of AF in men (HR 1.35, 95% CI 1.18 to 1.54) and 23% in women (HR 1.23, 95% CI 1.07 to 1.41).^26^ Tedrow *et al* reported a 41% higher risk for women who became obese compared with those who did not (HR 1.41, 95% CI 1.05 to 1.90).^24^

#### Weight gain by baseline weight

Two studies presented subgroup analyses by baseline weight. Grundvold *et al* reported weight gain in those who were obese at baseline was associated with a similar increase in risk of AF compared with the whole population (obese subgroup HR 1.59, 95% CI 1.02 to 2.47, whole population HR 1.53, 95% CI 1.10 to 2.12).^22^ Johnson *et al* reported weight gain of 1 kg per annum resulted in a similar increased risk of AF in people with healthy weight (BMI<25 kg/m^2^) compared with the whole study population (healthy BMI subgroup HR 1.13, 95% CI 0.99 to 1.30, whole population HR 1.12, 95% CI 1.03 to 1.23).^25^

### Weight loss and risk of incident AF

#### Percentage weight loss

In the RCT, the intervention group achieved a mean percentage weight loss of 6.0%, compared with 3.5% in the control group, and the HR for the onset of AF was 0.99 (95%CI 0.77 to 1.28).^21^ As results were reported by trial arm, those who did not lose weight were included in the analysis and therefore the results may underestimate any association between weight loss and AF.

**Figure 3:**
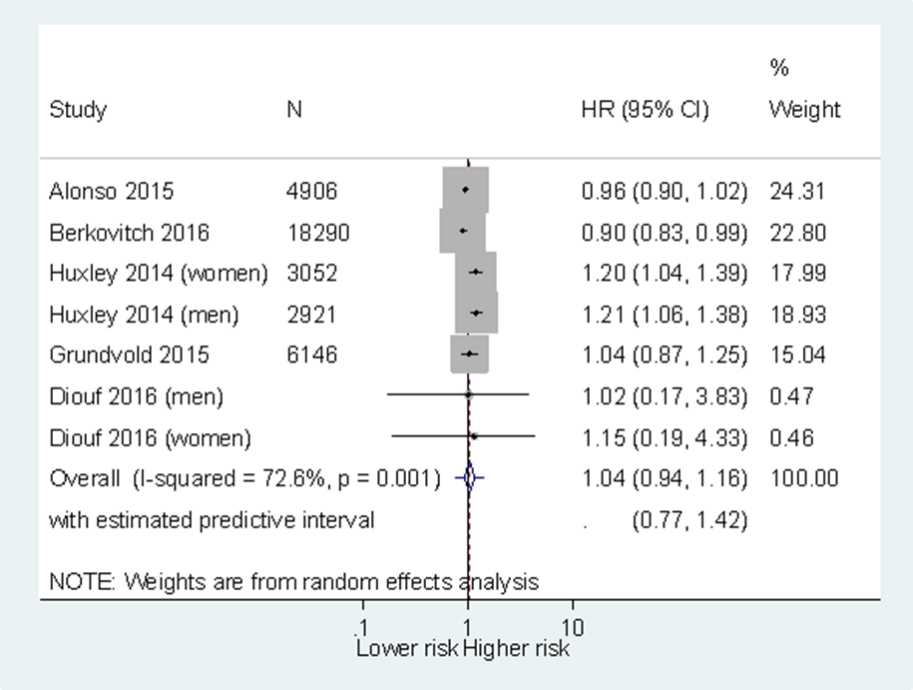
Risk of 5% weight loss on incidence of atrial fibrillation.

In a pooled analysis from seven populations (five studies), there was no statistically significant association between 5% weight loss and incidence of AF (HR 1.04, 95% CI 0.94 to 1.30 with a prediction interval 0.77 to 1.42) (figure 3). In all the studies, most participants were overweight or obese at baseline. Grundvold *et al* reported no significant difference in risk of AF between those who were obese at baseline and lost weight compared with the whole population in the only such subgroup analysis.^22^

There was high statistical heterogeneity (I^2^=73%). Excluding the study by Huxley *et al* 30 dropped the I^2^ to 0% and resulted in a minor but significant reduction in incidence of AF (HR 0.95, 95% CI 0.90 to 0.99) (eTable 3). In both studies of general populations and in the single study of a population with T2DM, there was no significant association between weight loss and AF. In studies with follow-up less than 10 years, there was a significant reduction in risk of AF and in the study populations with follow-up more than 10 years, there was a significant increase in risk (eTable 3).

10.1136/heartjnl-2019-314931.supp6 Supplementary file 6 table 3

Rosengren *et al* found no evidence that losing more than 4% body weight from age 20 years to midlife was associated with a significant change in the incidence of AF compared with individuals whose weight only changed by between −4% and 4% in the same period (HR 1.00, 95% CI 0.72 to 1.27).^29^ These results could not be pooled.

#### Other measures of weight loss

Ball *et al* reported a reduction in BMI of 2 kg/m^2^ was associated with a 18% decrease in risk of AF in men (HR 0.82, 95% CI 0.75 to 0.90) and 19% in women (HR 0.81, 95% CI 0.71 to 0.94), compared with people whose BMI increased by 1 kg/m^2^.^26^ Tedrow *et al* reported that women who were obese at baseline but lost weight to reduce their BMI below 30 kg/m^2^ no longer had an elevated risk of AF compared with people who were never obese (HR 1.01, 95% CI 0.58 to 1.79).^24^

#### Amount of weight loss and risk of AF

Four studies reported the impact of degrees of weight loss on the risk of AF, but the results were inconclusive.^21 27 30^ Alonso *et al* and Diouf *et al* both reported no significant association between weight loss and AF across all reported ranges of weight loss.^21 27^ Ball *et al* reported a non-significant trend towards a decrease in the risk of incident AF for people whose BMI decreased by up to 2 kg/m^2^ compared with those who gained 1 kg/m^2^.^26^ Huxley *et al* reported a significant increase in incident AF in those who lost more than 5% of their weight and suggested that conditions associated with muscle wasting, which will lead to unintentional weight loss, may explain their finding.^30^

### Quality assessment

The NOS scores ranged from 5 to 7 reflecting a moderate to low risk of bias (table 3). Based on the GRADE framework, there was moderate certainty in the 5% weight gain summary finding and very low certainty in the summary estimate of 5% weight loss. Further details of the quality assessments are included online (eResults).

10.1136/heartjnl-2019-314931.supp7 Supplementary file 7 result

**Table 3:** Risk of bias measured by the Newcastle-Ottawa Scale

Study lead author	Study year	Selection	Comparability	Outcome	Overall score (number of stars)
Rosengren *et al* 29	2009	****		**	6
Tedrow *et al* 24	2010	***		***	6
Grundvold *et al* 23	2012	***	*	**	6
Huxley *et al* 30	2014	****		***	7
Alonso *et al* 21	2015	**	*	***	6
Diouf *et al* 27	2015	***		**	5
Grundvold *et al* 22	2015	***		***	6
Johnson *et al* 25	2015	****		***	7
Berkovitch *et al* 28	2016	****		***	7
Ball *et al* 26	2018	****		**	6

Scores range from 0 to 9 stars with nine stars equating to the lowest risk of bias.

## Discussion

### Main findings

Weight gain was associated with an increased risk of AF in our pooled and unpooled analyses. Weight loss was not associated with an altered risk of incident AF in our pooled analysis. One unpooled study reported a significant association between weight loss and reduced AF incidence, another showed no association, and a third showed that those who dropped weight below the obesity threshold were no longer at an elevated risk of AF.^24 26 29^ In both pooled analyses, the majority of people were overweight or obese at baseline, although there were significant numbers of people with a normal BMI in most studies. Both results had high heterogeneity. There was moderate certainty in the weight gain summary finding, but only very low certainty in the weight loss finding.

### Comparison with other studies

Our results suggest that weight gain itself in addition to BMI status may be linked to future risk of AF. Three systematic reviews have showed that obesity increases the risk of incident AF.^7–9^ One also analysed weight gain and AF, reporting no significant association between a 5% increase in bodyweight and AF incidence, based on two studies (relative risk of AF 1.08, 95% CI 0.97 to 1.19).^7^ Our review expands on this result, adding three further studies in the pooled analysis.

### Strengths

This is the first systematic review to assess the risk of incident AF in relation to weight loss and extends our understanding of the association between weight gain and AF risk. We analysed data from 108 996 people across 10 individual studies, including seven in the pooled analyses. Most studies were conducted within the past 10 years across a range of economically developed countries and recruited middle-aged participants with a relatively low burden of comorbidity, providing contemporary, generalisable results.

### Limitations

Pooling data from people who were healthy weight with those who were obese or overweight at baseline may have diluted the summary estimates and thus underestimate the effect of weight loss on the subsequent risk of AF. Most studies had significant numbers of people who had a normal BMI at baseline and there were insufficient data to perform the planned subgroup analysis by baseline weight. The weight gain studies which did split by baseline weight status reported very similar estimates of AF risk across groups. Some studies reported AF incidence during follow-up after the period when weight change was measured and it is possible that further weight change may have occurred that altered the subsequent risk of AF. There was inconsistency in the results of our analysis of weight loss measured by percentage change and change in BMI, and we were unable to summarise the overall effect as it was not possible to pool all these data. Studies presented data differently, which we sought to overcome by making conservative approximations to convert data into a common form to enable pooling from more studies.

Some studies relied on periodic ECG assessments, which may miss cases of paroxysmal AF and so underestimate true incidence, but this is unlikely to have biased differences by weight change status. Many studies attempted to triangulate their case detection with other electronic health records. Two studies calculated weight change using a recalled weight as the baseline measure, but these results were consistent with other studies.^29^

Nine of our 10 studies were observational. We included results from multivariate analyses but relevant covariates such as physical activity and history of cardiovascular disease were sometimes excluded.

### Policy implications

Weight gain of 5% was associated with an increase in the risk of AF of 13% (95% CI 4% to 23%), with the likely effect in an individual setting varying between 18% reduction and a 44% increase in risk. Our results suggest that the risk of incident AF continues to rise in relation to weight gain across all categories of weight status. This evidence supports public health interventions that promote maintaining a healthy weight and preventing weight gain to reduce the burden of AF. Given the paucity of data and high heterogeneity, our conclusions are only tentative.

Whether intentional weight loss can reduce the risk of developing AF remains uncertain and may be related to the amount of weight people lose.^31^ Large amounts of weight loss achieved through bariatric surgery has been reported to reduce the risk of AF by 29% compared with no weight change (HR 0.71, 95% CI 0.60 to 0.83) over long-term follow-up.^32^

Unintentional weight loss may occur as a result of diseases that are independent risk factors for AF, such as cancer, hyperthyroidism or muscle wasting conditions as suggested by one of the included studies, which reported weight loss being associated with a significant increased risk of developing AF.^30^

### Future research

Obesity is the second highest population attributable risk factor for incident AF behind hypertension, accounting for over 10% of all cases.^33^ The link between obesity and incident AF has been established, but more research is needed to understand the degree of increased risk associated with weight gain, whether non-surgical weight loss can reduce the risk of incident AF and if this depends on baseline weight status, intentionality and amount of weight loss.

## Conclusion

Gaining weight increases the risk of incident AF, but the evidence is uncertain whether relatively small amounts of non-surgical weight loss reduces that risk. Interventions that help prevent weight gain in both individuals and populations, even in those already overweight, may reduce the growing global burden of AF and related healthcare costs.

**Key messages**

**What is already known on this subject?**

- While there is an established association between obesity and an increase in the incidence of atrial fibrillation (AF), the relationship between weight change and the risk of AF is unclear.

**What might this study add?**

- In this systematic review and meta-analysis of over 100 000 people, we found weight gain was associated with an increase in the incidence of AF, but no consistent association between non-surgical weight loss and altered incidence of AF.

**How might this impact on clinical practice?**

- Preventing weight gain could be an important strategy in reducing the global burden of AF.
