# Supplementary material for: Weight change and the risk of incident atrial fibrillation: a systematic review and meta-analysis
Source: Heart. 2019 Jun 22;105(23):1799–805. doi: 10.1136/heartjnl-2019-314931 (PMC6900224; doi:10.1136/heartjnl-2019-314931)
Supplement: Supplementary file 1 [file heartjnl-2019-314931supp001.docx]

**eMethods 1. Study protocol**

This protocol was completely in advance of data extraction but not prospectively registered

**A systematic review and meta-analysis of the association between weight change and the risk of incident atrial fibrillation**

Nicholas R Jones, Kathryn Taylor, Paul Aveyard

University of Oxford Nuffield Department of Primary Care Health Sciences, Radcliffe Primary Care Building, Radcliffe Observatory Quarter, Woodstock Rd, Oxford OX2 6GG

**Background**

Atrial fibrillation (AF) is the most common sustained cardiac arrhythmia, affecting around 1 million people in the UK and 5 million worldwide [1,2]. It is linked to a number of significant negative outcomes, including embolic stroke (relative risk 2.42, 95% CI 2.17-2.71) and all-cause mortality (1.46, 1.39-1.54) [3]. AF is also associated with reduced quality of life, impacting on physical and social functioning as well as mental health [4,5]. The prevalence of AF is increasing with reported cases doubling in Europe in the past 10 years and recent estimates suggesting there will be between 120,000-215,000 new cases per year in Europe by 2030 [6]. Potential reasons include an ageing population, improved outcomes following acute coronary events, improved detection of AF and the rising prevalence of obesity.

The increasing prevalence of overweight or obese individuals in more economically developed countries has been well documented: In England in 2014, just over 60% of adults were overweight or obese and there was a rise in obesity prevalence from around 15% to 25% between 1993 and 2014 [9]. Obesity negatively affects electrical and structural cardiac function because of increased epicardial adipose tissue, atrial enlargement and diastolic dysfunction resulting in atrial remodelling [7]. Obesity is also associated with other diseases that are independent risk factors for AF including hypertension, type 2 diabetes mellitus and obstructive sleep apnoea. There is an increased risk of AF in people who are overweight (relative risk 1.39, 95%CI 1.05-1.83) and obese individuals (relative risk 1.87 95% CI 1.43-2.44 compared to those of healthy weight [8].

Some studies have generally suggested that weight gain increases the risk of developing AF but with less clear associations reported between weight loss and the incidence of AF. There is no up-to-date systematic review on the topic. This information is important to establish, as understanding the impact of weight change on the risk of developing AF could inform and improve future preventative management and public health measures. Obesity may be treated more proactively if the subsequent healthcare benefits can be proven, the results may provide additional motivation for weight loss in people at higher risk of developing AF, leading to a reduced incidence and preventing complications, such as strokes.

**Review question**

We will assess the strength and direction of the association between change in weight and the incidence of AF. As we expect the direction of association may vary, we will split studies into those where the exposure was weight loss and those where it was weight gain. We will examine whether the direction of association is modified by the starting weight status of the population.

**Methods**

Eligibility criteria

Study design: Our scoping searched revealed no randomized trials of weight loss interventions that had reported on the incidence of AF as an outcome and obviously, no such trials would be possible addressing weight gain. We therefore will include cohort studies. There will be no limitation on the number of participants or duration of follow-up.

Setting: Any country and any healthcare setting. Any language. Human studies only. No limitations on publication date.

Publication type: Primary studies. Case reports and case series will be excluded because they provide no data on the incidence of AF. Conference abstracts will only be included if they are related to a more detailed publication article. Conference abstracts alone are excluded as lacking insufficient detail in methodology and results.

The review will be based around the following PICO:

Population: Adults, aged 18 years or older, as AF is extremely rare in children and likely to have a different pathophysiology. Participants may be at any weight and will be without atrial fibrillation at the study outset but may have other cardiovascular history or risk factors.

Exposure variable: Weight change is the exposure variable. It may be reported as a change in body mass index (BMI), weight or waist circumference. Both intentional and unintentional weight change will be considered. We will seek cohorts where the investigators had data on whether or not participants were intentionally losing weight but we recognize that such data are rare and will include those cohorts where this information is not available. Where weight loss is induced through a weight loss intervention, we will analyse data by trial arm where this is possible but keep the data separate from the observational data. If the same study reports on the incidence of AF by achievement of weight loss, then these data will be included in the observational analyses. Where there is no data on intentionality of weight loss, it is possible that this weight loss is induced by another underlying illness, which could be a confounding cause of AF. For example, a patient who develops cancer or thyroid disease may lose significant amounts of weight but also be at a higher risk of developing AF. Consequently, we will do subgroup analysis by intentionality of weight loss. We will also plan to do subgroup analysis by original weight status as final weight status will be related to this and may impact on the risk of incident AF.

Outcome: The incidence of paroxysmal, permanent or persistent AF

**Search strategy**

The following databases will be searched: Medline, Embase, Cinahl, Cochrane Central Register of Controlled Trials, Database of Abstracts of Reviews of Effects, Web of Science and Trial Registers - [clinicaltrials.gov](http://clinicaltrials.gov) and WHO ICTRP. The literature search will be developed in collaboration with a departmental librarian who is experienced in systematic reviews. Free text and MESH headings (atrial fibrillation and body weight change) will be used.

**Screening and data extraction**

One reviewer will screen all papers from the search for eligibility, as per the pre-defined criteria, first via title and abstract, and then via the full text. A second reviewer will verify and agree the final list of included papers. Study selection will be summarized in a PRISMA flow diagram.

A data extraction form will be used to record demographic data (including age, gender, weigh status at baseline, co-morbidities and study details (including healthcare setting, country of origin, study design, follow-up time, intervention, method and frequency of screening for incident AF) and outcome. In a protocol update in July 2018 we decided to exclude studies looking at risk of recurrent AF following previous ablation, as we felt the risk in this group was likely to be different to that of the general population. Two authors will extract the data.

**Risk of bias**

The shortlisted papers will be independently assessed by two reviewers for risk of bias using the Newcastle Ottawa risk of bias scale [10]. This tool covers potential biases in the studies owing to selection bias (randomisation, allocation), blinding (participants and/or outcomes), attrition, and selective reporting. Any disagreement will be discussed with a third reviewers to reach consensus. A GRADE assessment is also planned to assess for risk of bias between studies and the certainty in the summary findings.

**Data analysis and synthesis**

All data analyses will be carried out using STATA v14. We will produce summary tables of descriptive statistics of the patient and study characteristics. Appropriate statistical methods will be applied to convert the data from all included studies into a common format, in preparation for pooling, and random effects meta-analysis will be carried out, based on the DerSimonian and Laird method [11]. Results will be expressed as relative risks with 95% confidence intervals and displayed in forest plots. Heterogeneity will be assessed using the I^2^ statistic. The potential sources of any heterogeneity (clinical or statistical) will be explored. Where the heterogeneity is very high (>75%) and any pooling of data would be considered to be misleading, the results will be presented in a narrative form. Data which cannot be pooled will be presented in a narrative form.

Subgroup analyses by gender, co-morbidity, initial weight and by intention of weight loss will be carried out if possible. Studies will be excluded, one-by-one, as part of a sensitivity analysis. Random-effects meta-regression will be carried out to evaluate the explanatory value of co-morbidities if there are sufficient data.

**References**

1. Chugh SS, Havmoeller R, Narayanan K, et al. Worldwide epidemiology of atrial fibrillation: a Global Burden of Disease 2010 Study. *Circulation*2014;129:837-47
2. National Institute for Health and Care Excellence Clinical guideline [CG180] Atrial fibrillation: management. June 2014
3. Odutayo A, Wong C, Hsiao AJ, Hopewell S, Altman DG, Edin CA. Atrial fibrillation and risks of cardiovascular disease, renal disease, and death: systematic review and meta-analysis *BMJ* 2016;354:i4482
4. Dorian P, Jung W, Newman D, Paquette M, Wood K, Ayers GM, Camm J, Akhtar M, Luderitz B. The impairment of health-related quality of life in patients with intermittent atrial fibrillation: implications for the assessment of investigational therapy *J Am Coll Cardiol*. 2000;36(4):1303-1309.
5. Thrall G, Lane D, Carroll D, Lip GY. Quality of life in patients with atrial fibrillation: a systematic review. *Am J Med* 2006;119:448.e1-19
6. [Zoni-Berisso M](https://www.ncbi.nlm.nih.gov/pubmed/?term=Zoni-Berisso%2525252520M%252525255BAuthor%252525255D&cauthor=true&cauthor_uid=24966695), [Lercari F](https://www.ncbi.nlm.nih.gov/pubmed/?term=Lercari%2525252520F%252525255BAuthor%252525255D&cauthor=true&cauthor_uid=24966695), [Carazza T](https://www.ncbi.nlm.nih.gov/pubmed/?term=Carazza%2525252520T%252525255BAuthor%252525255D&cauthor=true&cauthor_uid=24966695), [Domenicucci S](https://www.ncbi.nlm.nih.gov/pubmed/?term=Domenicucci%2525252520S%252525255BAuthor%252525255D&cauthor=true&cauthor_uid=24966695). Epidemiology of atrial fibrillation: European perspective. [Clin Epidemiol.](https://www.ncbi.nlm.nih.gov/pubmed/24966695) 2014 Jun 16;6:213-20.
7. [Goudis CA](https://www.ncbi.nlm.nih.gov/pubmed/?term=Goudis%2525252520CA%252525255BAuthor%252525255D&cauthor=true&cauthor_uid=25959929), [Korantzopoulos P](https://www.ncbi.nlm.nih.gov/pubmed/?term=Korantzopoulos%2525252520P%252525255BAuthor%252525255D&cauthor=true&cauthor_uid=25959929), [Ntalas IV](https://www.ncbi.nlm.nih.gov/pubmed/?term=Ntalas%2525252520IV%252525255BAuthor%252525255D&cauthor=true&cauthor_uid=25959929), [Kallergis EM](https://www.ncbi.nlm.nih.gov/pubmed/?term=Kallergis%2525252520EM%252525255BAuthor%252525255D&cauthor=true&cauthor_uid=25959929), [Ketikoglou DG](https://www.ncbi.nlm.nih.gov/pubmed/?term=Ketikoglou%2525252520DG%252525255BAuthor%252525255D&cauthor=true&cauthor_uid=25959929) Obesity and atrial fibrillation: A comprehensive review of the pathophysiological mechanisms and links. [J Cardiol.](https://www.ncbi.nlm.nih.gov/pubmed/25959929) 2015 Nov;66(5):361-9.
8. Wanahita N, Messerli FH, Bangalore S, Gami AS, Somers VK, Steinberg JS. Atrial fibrillation and obesity - results of a meta-analysis. *Am Heart Jour* 2008 Feb. 155;2: 310-315
9. Public health England <https://www.noo.org.uk/NOO_about_obesity/adult_obesity/UK_prevalence_and_trends>
10. Wells G, Shea B, O’Connell D, Peterson J, Welch V, Losos M, Tugwell P. The Newcastle-Ottawa Scale (NOS) for assessing the quality of nonrandomised studies in meta-analyses. 2013. <http://www.ohri.ca/programs/clinical_epidemiology/oxford.asp>.
11. Higgins JP, Altman DG, Gøtzsche PC et al. The Cochrane Collaboration’s tool for assessing risk of bias in randomised trials BMJ 2011;343:d5928
12. DerSimonian R, Laird N. Meta-analysis in clinical trials. Control Clin Trials 1986; 7 : 177–188.
