# Supplementary material for: Weight change and the risk of incident atrial fibrillation: a systematic review and meta-analysis
Source: Heart. 2019 Jun 22;105(23):1799–805. doi: 10.1136/heartjnl-2019-314931 (PMC6900224; doi:10.1136/heartjnl-2019-314931)
Supplement: Supplementary file 8 [file heartjnl-2019-314931supp008.docx]

**eResults. Quality of evidence assessment**

Risk of bias within individual studies

The large sample sizes, often recruited from the general population, prolonged follow-up and the ability to exclude a pre-existing AF at baseline contributed to many studies having a relatively low risk of bias.

GRADE assessment

There was consistency in the direction of association between 5% weight gain and risk of incident of AF. Some studies reported a dose response gradient, with increasing weight gain related to increasing risk of AF.^28^ The studies predominantly recruited healthy volunteers meaning the results provide evidence applicable to the general public. The confidence in each summary finding was affected by the reliance on observational data and concerns around the precision of results, as the clinical relevance would alter significantly depending whether the truth lay closer to the upper or lower confidence interval. We were unable to test for the possibility of reporting bias as there were insufficient studies. For weight loss, there was unexplained inconsistency between studies in the direction of association between 5% weight loss and the risk of AF. Only one of the studies adequately controlled for intentionality of weight loss, a key potential confounder.
