# Supplementary material for: Weight change and the risk of incident atrial fibrillation: a systematic review and meta-analysis
Source: Heart. 2019 Jun 22;105(23):1799–805. doi: 10.1136/heartjnl-2019-314931 (PMC6900224; doi:10.1136/heartjnl-2019-314931)
Supplement: Supplementary file 3 [file heartjnl-2019-314931supp003.docx]

**eMethods 2. Search strategy**

Electronic databases (searched to July 2018)

MEDLINE, Embase, Pubmed, Web of Science, Cochrane Central Register of Controlled Trials, Database of Abstracts of Reviews of Effects, Trials Register - [clinicaltrials.gov](http://clinicaltrials.gov), CINAHL and the World Health Organisation (WHO) International Clinical Trials Registry Platform (ICTRP).

Search terms for MEDLINE (via OVID)

| 1 | atrial fibrillation.mp. or atrial fibrillation/ |
| --- | --- |
| 2 | body weight/ or weight/ or weight.mp. |
| 3 | body mass index.mp. or body mass/ |
| 4 | obesity/ or body mass |
| 5 | BMI/ |
| 6 | 2 or 3 or 4 or 5 |
| 7 | gain.mp. or weight gain |
| 8 | weight loss.mp. or weight reduction/ |
| 9 | weight loss program/ or loss.mp. |
| 10 | weight change/ |
| 11 | increas* |
| 12 | decreas* |
| 13 | 7 or 8 or 9 or 10 or 11 or 12 |
| 14 | inciden*.mp. or incidence/ |
| 15 | risk/ or risk factor/ or risk.mp. |
| 16 | develop.mp. |
| 17 | 14 or 15 or 16 |
| 18 | 1 and 5 and 21 and 25 |
| 19 | limit 26 to (human and journal and (adult<18 to 64 years> or aged <65+ years>) |
