# Supplementary material for: Weight change and the risk of incident atrial fibrillation: a systematic review and meta-analysis
Source: Heart. 2019 Jun 22;105(23):1799–805. doi: 10.1136/heartjnl-2019-314931 (PMC6900224; doi:10.1136/heartjnl-2019-314931)
Supplement: Supplementary file 4 [file heartjnl-2019-314931supp004.docx]

**eMethods 3. Contour plots of included studies for weight gain and weight loss**

Contour plots are funnel plots with shading superimposed to indicate the distribution of the statistical significance of studies.

Shading: White (p>10%); dark grey (5%<p<10%); medium grey (1%<p<5%); light grey (p<1%).

Positive logHR indicates increased risk of AF and negative logHR indicates a reduced risk of AF

3.1 Contour plot of studies included in pooled analysis of risk of 5% weight gain on incidence of atrial fibrillation

3.2 Contour plot of studies included in pooled analysis of risk of 5% weight loss on incidence of atrial fibrillation
