# Supplementary material for: Weight change and the risk of incident atrial fibrillation: a systematic review and meta-analysis
Source: Heart. 2019 Jun 22;105(23):1799–805. doi: 10.1136/heartjnl-2019-314931 (PMC6900224; doi:10.1136/heartjnl-2019-314931)
Supplement: Supplementary file 5 [file heartjnl-2019-314931supp005.docx]

**eTable 1. Potential confounders adjusted for in the multivariate analysis of each study**

| First author (year) | Categories of weight loss data | Confounders adjusted for in multivariate analysis |
| --- | --- | --- |
| Rosengren^29^ (2009)  [Table 4 – multiple adjusted HR] | No – loss >4% single group | Baseline age, intercurrent heart failure (time-dependent), intercurrent myocardial infarction (time-dependent), systolic blood pressure, treatment for hypertension, smoking, diabetes, alcohol problems, occupational class. |
| Tedrow^24^ (2010)  [Table 3 – multivariable adjusted HR] | No – single group who were obese and then reduced BMI to <30 | Age, BMI, race, vitamin E, beta carotene, aspirin, diabetes, hypertension, hyperlipidaemia, alcohol use, smoking and degree of physical activity. |
| Grundvold^23^ (2012)  [Table 6 – multiple adjusted HR] | No – weight loss single group | Age, systolic blood pressure, current smoking status, total cholesterol and blood glucose. |
| Huxley^30^ (2014)  [Sup table 3] | Yes 0-5% and >5% weight loss groups. Increasing risk of AF with greater weight loss | Age, race, study site, education, income, prior cardiovascular disease, height, cigarette smoking, physical activity and alcohol consumption. |
| Alonso^21^ (2015)  [Table IV – model 3] | Yes, four categories of weight loss | Clinic, age, sex, race, intervention group, family income, smoking, BMI, height, systolic blood pressure, diastolic blood pressure, use of antihypertensive medication, HbA1c, prevalent coronary heart disease and prevalent heart failure. |
| Grundvold^22^ (2015)  [Table 3 - model B] | No – single weight loss group. Did have subgroup analysis by obese at baseline | Age, gender, BMI at baseline, previous angina pectoris, systolic blood pressure. |
| Johnson (2015)^25^  [Table 2 – model 2^b^] | No | Age, weight, height, systolic blood pressure, fasting blood glucose, smoking status, sedentary lifestyle, screening year, prevalent anti-hypertensive medication at baseline, anti-hypertensive medication initiated before rescreening, low socioeconomic index, FEV1, alcohol use and baseline data of the delta variable of interest. |
| Berkovitch^28^ (2016)  [In text, statistical analysis section] | No | Age, gender, baseline BMI, baseline LDL and baseline low HDL cholesterol plus ischaemic heart disease, hypertension and diabetes mellitus as time-dependent covariates. |
| Diouf^27^ (2016)  [Figure 1] | Data extracted from percentage weight change graphs | Age, gender, BMI, smoking status, usual number of alcoholic drinks, physical activity and level of education. |

BMI – body mass index; FEV1 - forced expiratory volume in 1 second; HDL – high density lipoprotein; LDL – low density lipoprotein
