# Supplementary material for: Weight change and the risk of incident atrial fibrillation: a systematic review and meta-analysis
Source: Heart. 2019 Jun 22;105(23):1799–805. doi: 10.1136/heartjnl-2019-314931 (PMC6900224; doi:10.1136/heartjnl-2019-314931)
Supplement: Supplementary file 6 [file heartjnl-2019-314931supp006.docx]

**eTable 2. Sensitivity analysis results for studies of percentage weight gain**

|  | Populations  (studies) | Hazard ratio | 95% confidence interval | | I^2^ |
| --- | --- | --- | --- | --- | --- |
| Random effects model (base case) | 6 (5) | 1.128 | 1.037 | 1.227 | 69.6% |
| *SENSITIVITY ANALYSIS* |  |  |  |  |  |
| Fixed effects model | 6 (5) | 1.047 | 1.025 | 1.069 | 69.6% |
| Excluding studies with estimates  (Grundvold 2012 and 2015) | 4 (3) | 1.115 | 0.989 | 1.259 | 70.3% |
| Excluding Huxley 2014 | 4 (4) | 1.091 | 1.000 | 1.19 | 59.3% |
| Excluding Diouf 2016 | 5 (4) | 1.128 | 1.035 | 1.229 | 75.2% |
| Excluding Rosengren 2009 | 5 (4) | 1.165 | 1.071 | 1.268 | 33.1% |
| Excluding Grundvold 2012 | 5 (4) | 1.152 | 1.018 | 1.303 | 73.1% |
| Excluding Grundvold 2015 | 5 (4) | 1.103 | 1.020 | 1.194 | 66.1% |
| Pooling data for Huxley 2014 | 5 (5) | 1.107 | 1.020 | 1.201 | 59.3% |
| *SUBGROUP ANALYSIS* |  |  |  |  |  |
| General populations only | 5(5) | 1.103 | 1.020 | 1.194 | 66.1% |
| Type 2 diabetes only (Grundvold 2015) | 1 | 1.340 | 1.069 | 1.678 | - |
