# Supplementary material for: Weight change and the risk of incident atrial fibrillation: a systematic review and meta-analysis
Source: Heart. 2019 Jun 22;105(23):1799–805. doi: 10.1136/heartjnl-2019-314931 (PMC6900224; doi:10.1136/heartjnl-2019-314931)
Supplement: Supplementary file 7 [file heartjnl-2019-314931supp007.docx]

**eTable 3. Sensitivity analysis results for studies of percentage weight loss**

|  | Populations  (studies) | Hazard ratio | 95% confidence interval | | I^2^ |
| --- | --- | --- | --- | --- | --- |
| Random effects model (base case) | 7 (5) | 1.043 | 0.937 | 1.162 | 72.6% |
| *SENSITIVITY ANALYSIS* | | | | | |
| Fixed effects model | 7 (5) | 0.998 | 0.990 | 1.007 | 72.6% |
| Excluding studies with estimates  (Grundvold 2015 and Berkovitch 2016) | 5 (3) | 1.006 | 0.941 | 1.300 | 73.6% |
| Excluding Huxley 2014 (weight gain ref group) | 5(4) | 0.946 | 0.901 | 0.992 | 0.0% |
| Excluding Diouf 2016, men (concerns about data) | 6(5) | 1.044 | 0.935 | 1.166 | 77.2% |
| Excluding Diouf 2016, men and women | 6 (5) | 1.044 | 0.932 | 1.170 | 81.7% |
| Excluding Alonso 2015 | 6(4) | 1.076 | 0.920 | 1.258 | 74.4% |
| Excluding Berkovitch 2016 | 6(4) | 1.090 | 0.960 | 1.238 | 67.0% |
| *SUBGROUP ANALYSIS* | | | | | |
| General populations only | 5 (3) | 1.088 | 0.891 | 1.328 | 79.7% |
| Type 2 diabetes populations only | 2 | 0.968 | 0.912 | 1.026 | 72.6% |
